# A platform for stereological quantitative analysis of the brain-wide distribution of type-specific neurons

**DOI:** 10.1038/s41598-017-14699-w

**Published:** 2017-10-30

**Authors:** Chen Zhang, Cheng Yan, Miao Ren, Anan Li, Tingwei Quan, Hui Gong, Jing Yuan

**Affiliations:** 1 Britton Chance Center for Biomedical Photonics, Wuhan National Laboratory for Optoelectronics-Huazhong University of Science and Technology, Wuhan, Hubei 430074 China; 2 MoE Key Laboratory for Biomedical Photonics, Collaborative Innovation Center for Biomedical Engineering, School of Engineering Sciences, Huazhong University of Science and Technology, Wuhan, Hubei 430074 China

## Abstract

Quantifying the distribution of specific neurons throughout the whole brain is crucial for understanding physiological actions, pathological alterations and pharmacological treatments. However, the precise cell number and density of specific neurons in the entire brain remain unknown because of a lack of suitable research tools. Here, we propose a pipeline to automatically acquire and analyse the brain-wide distribution of type-specific neurons in a mouse brain. We employed a Brain-wide Positioning System to collect high-throughput anatomical information with the co-localized cytoarchitecture of the whole brain at subcellular resolution and utilized the NeuroGPS algorithm to locate and count cells in the whole brain. We evaluated the data continuity of the 3D dataset and the accuracy of stereological cell counting in 3D. To apply this pipeline, we acquired and quantified the brain-wide distributions and somatic morphology of somatostatin-expressing neurons in transgenic mouse brains. The results indicated that this whole-brain cell counting pipeline has the potential to become a routine tool for cell type neuroscience studies.

## Introduction

The brain is an important and extremely complex organ. In 1888, Santiago Ramón y Cajal revolutionarily modified Golgi’s method to stain neurons^1^; since then, the neuroscience field has developed for more than a century. Magnetic resonance imaging and electron microscopy have helped us understand the brain at the macroscopic and microscopic levels, respectively. However, the large range and fine structure of the brain present great challenges to deciphering fundamental brain structure at cellular resolution. Suitable research tools and adequate neuroanatomical data are lacking, and the precise number, morphology, connectivity and type of neurons in the brain remain unsolved mysteries in neuroscience^2^.

The identification and quantification of specific-type neurons yields insights into the fine structural and functional organization of specific brain regions and provides the basis for precise functional manipulations. Soma location and cell density of specific-type neurons are strong predictors of cell subtype^3^. Identification of important features, including the relationship within soma location, cell density, dendritic and axonal morphology, connectivity, physiology and transcriptional profiles, will reveal detailed information of sub-type neurons. Furthermore, the spatial distribution and morphological features of neuronal cell bodies in specific brain regions show significant variations in developmental stage and various physiological and pathological states^4–9^. Locating, counting and reconstructing type-specific neurons will facilitate our understanding of brain functions and diseases. Changes in neuronal density, decrease in volume and reductions in neuronal size are quantitative aspects of brain disease diagnosis^8,10–12^. However, traditional histology relies on manual approaches to obtain anatomical information, and the manual generation and imaging of serial sections is laborious and time consuming. Limited by the tremendous number of neurons throughout the brain, cell numbers have been estimated by interval sampling whole-brain slices; however, this method leads to clearly inaccurate data. Planar cell counting (in two dimensions, 2D) in maximum intensity projections for each slice create potential errors due to the overlap of aligned neurons along the z-axis^13–16^. Furthermore, cells located on the interfaces of adjacent slices might be counted twice. Therefore, it is urgent to develop a novel research tool for stereological cell counting (in three dimensions, 3D) to address the fundamental neuroscience question of total cell numbers in the brain.

The recent developments of innovative fluorescence labelling, optical imaging and imaging processing have enabled the assessment of the cellular contribution of type-specific cells in 3D neuronal networks^17^. Genetic^18,19^ and viral^20^ techniques unambiguously target specific cell types according to the molecular expression of functional proteins in the brain. Fluorescently labelled proteins in targeted neurons create contrast and specificity for imaging. High-throughput whole-brain optical imaging techniques have collected 3D images of the whole rodent brain at micron-scale resolution and shows potential to describe the cell distribution in particularly rich detail. Light-sheet illumination microscopy (LSIM)^21^ employs chemical clearing^22^ to expand the imaging range to the entire mouse brain. Benefiting from high-throughput imaging, this technology is capable of imaging a cleared brain in less than one day. However, it is difficult to resolve nearby cell bodies within the entire imaging volume, which is limited by inevitable residual tissue scattering. Serial two-photon (STP) tomography^23^ and micro optical-sectioning tomography (MOST)^24^ combine optical microscopy with automatic mechanical sectioning to overcome the depth limitation of optical imaging, mapping neurons and neuronal wirings in the whole brain. To shorten the data acquisition time, STP tomography images the brain using rough axial sampling and obtains serial coronal images with a loss of data continuity. In contrast, fluorescence MOST serial technology achieves uniform and continuous whole-brain imaging, enabling mouse brain reconstruction with high contrast and resolution in 3D to unambiguously resolve fluorescently labelled neurons^25–27^. In particular, Brain-wide Positioning System (BPS)^27^ immersed the sample in a propidium iodide (PI) solution during data acquisition and employed the interval between sectioning and imaging to counterstain co-located cytoarchitecture for fluorescence-labeled neurons in real time. This method provides an accurate anatomical reference at single-neuron resolution for studying the pattern characteristics of neural structures in the brain. In addition, many computational techniques have been developed to automatically extract and quantify human-readable information from the whole-brain dataset. Automatic localization and segmentation of cell bodies from the image stacks^28–31^ are used to obtain an accurate count of numerous neurons. Among these methods, NeuroGPS is robust to the diversity of size, morphology and density of labelled neurons^28^. Especially, considering the distribution sparseness of neuronal positions in 3D space, NeuroGPS constructed an L1-minimization model to locate somas. This consideration enabled NeuroGPS to eliminate the interference from neurites on soma’s localization, which was a key factor for generating the trustworthy localization’s results. The availability of advanced labelling, imaging and computing techniques for whole-brain cell counting creates a new opportunity to systematically characterize the distribution and number of cell types. Silvestri, L. *et al*.^17^ applied light sheet microscopy and automated cell-identification algorithm to characterize the spatial distribution of Purkinje cells (PCs) across the whole cerebellum. However, a comprehensive quantification of brain-wide and regional distributions of type-specific neurons by whole-brain imaging and automated imaging analysing hasn’t been demonstrated.

Here, we propose a platform to analyse the brain-wide distribution of type-specific neurons from a 3D high-resolution fluorescence MOST dataset. This platform combined whole-brain optical imaging using the BPS and stereological localization using NeuroGPS to generate an accurate stereological cell count in the whole mouse brain. To validate our platform, we mapped and quantified the whole-brain distribution pattern of SOM-expressing neurons, an important subset of GABAergic interneurons^32^. SOM receptors are widely expressed in the central nervous system, particularly in the neocortex^33^ and hippocampus^34^. SOM is involved in many brain functions, including sensory, activity, sleep and cognitive processes^35^. SOM neurons inhibit neuronal activation and suppress neuronal firing^36^. Previous studies have comprehensively analysed the electrophysiological features, firing properties, local connectivity and regulatory functions of SOM neurons in different brain regions and have divided them into various subsets^37,38^. However, the precise whole-brain and regional distribution of SOM neurons have not been quantitatively described. To demonstrate the power of this platform, we obtained high-resolution brain-wide datasets for SOM-IRES-Cre:Ai3-EYFP mouse brains and quantitatively analysed the distribution patterns of somatostatin (referred to as SOM in this manuscript and referred to as SST) expression and characterized the morphological features of SOM neurons in the whole brain.

## Results

### Validation of the SOM-Cre mouse line

**Figure 1:**
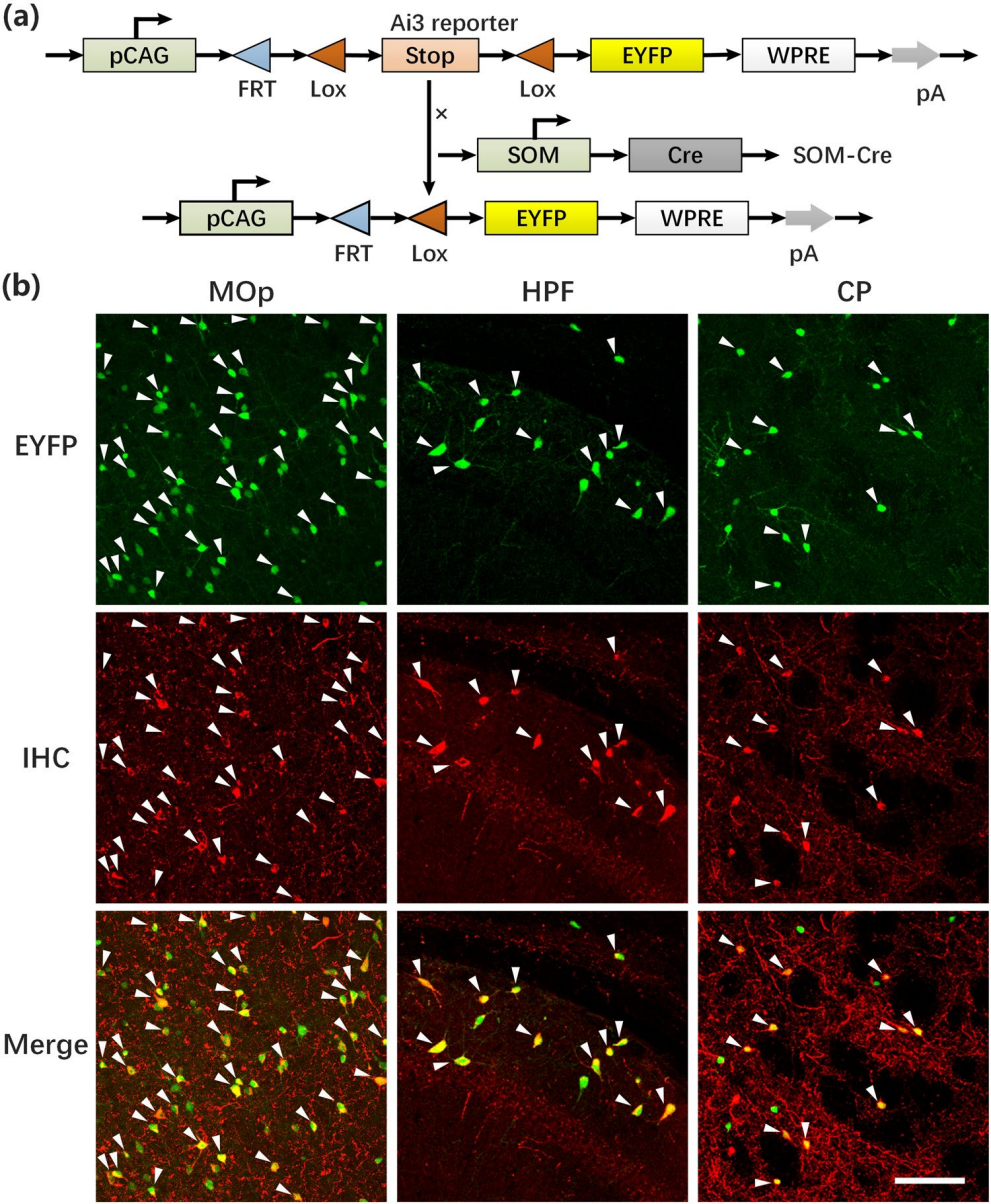
Distribution of EYFP-labelled interneurons and specific co-localization with SOM. (**a**) Gene elements of the Ai3 Cre-reporter mouse and SOM-Cre-line mouse used in the experiments. (**b**) Representative examples of specific co-localization of EYFP-labelled neurons (top, green) from a SOM-IRES-Cre:Ai3-EYFP mouse with immunofluorescence for somatostatin (middle, red). Merged images are shown at the bottom. Scale bars, 100 µm. Nearly all of the EYFP-labelled neurons from SOM-IRES-Cre:Ai3-EYFP mice co-localized with somatostatin in MOp, HPF and CP. Abbreviations: MOp, Primary motor area; HPF, Hippocampal formation and CP, Caudoputamen. SOM-positive rates in MOp, HPF and CP were 74.27 ± 1.31% (n = 3), 80.80 ± 1.39% (n = 3) and 77.53 ± 2.19% (n = 3), respectively.

We first confirmed the accuracy of using the SOM-IRES-Cre:Ai3-EYFP transgenic mouse (Fig. 1a) to genetically target SOM neurons. We performed immunostaining using an antibody against SOM in sections from the primary motor area (MOp), the hippocampal formation (HPF) and the caudoputamen (CP) and collected images using a Zeiss 710 confocal microscope with a 20× objective (Fig. 1b). The overlapping images of fluorescently labelled somas and immunostained molecules showed that the majority of the enhanced yellow fluorescent protein (EYFP)-labelled neurons were SOM-positive (SOM+), consistent with previous studies^32,39^. These results indicated that SOM-IRES-*Cre* driver lines reliably target SOM neurons and can be used to study the distribution of all SOM neurons in the whole brain.

### Whole-brain optical imaging

**Figure 2:**
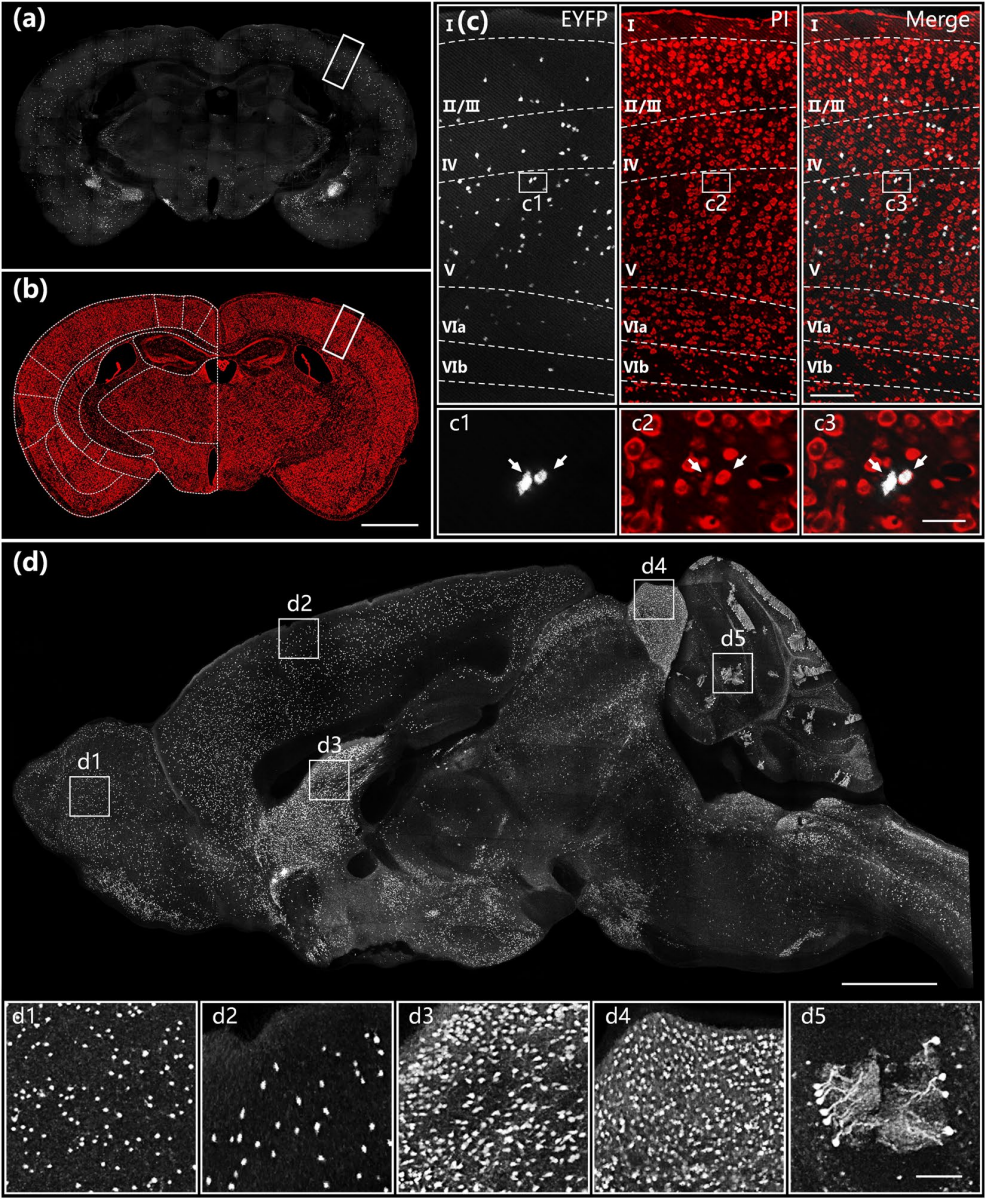
Whole-brain imaging of a SOM-IRES-Cre:Ai3-EYFP mouse brain. Images of EYFP-labelled neurons (**a**) and PI-stained cytoarchitecture (**b**) in the hippocampal coronal plane. (**c**) Enlarged views of white rectangular boxes indicated in (**a**) and (**b**) and their merged image. Image size is 900 × 400 μm. Projection thickness in the EYFP channel was 20 μm. (**d**) Sagittal reconstruction of maximum intensity projections of the SOM-IRES-Cre:Ai3-EYFP mouse brain. The projection thickness shown in (**d**) is 20 μm. The insets (d1)–(d5) are enlarged views of white square boxes shown in (**d**). The sizes of (d1)–(d5) are 400 × 400 × 20 μm. Scale bar: (**a**) and (**b**) 1 mm, (**c**) 25 μm, (c1)–(c3) 50 μm, (**d**) 1 mm and (d1)–(d5) 100 μm.

We acquired the whole-brain datasets from three SOM-IRES-Cre:Ai3-EYFP mice, including EYFP-labelled SOM neurons and PI-stained anatomical reference, in coronal planes (Fig. 2). Figure 2a and b show typical SOM distribution and co-localized cytoarchitecture in the hippocampal coronal plane, respectively. The PI-stained landmarks enabled us to easily identify the distinct laminar distribution of SOM neurons with different cell densities in different layers in a typical six-layer structure in the cortex in Fig. 2c. The overlap of GFP- and PI-channels suggested that a voxel resolution of 0.32 × 0.32 × 2 μm enables orientation of the neuronal cell bodies based on counterstained nuclei at single cell resolution (inset in Fig. 2c). The high resolution and data continuity of BPS facilitated brain reconstruction along the sagittal direction by resampling original coronal images at 0.32 × 0.32 × 2 μm. SOM cells were primarily distributed in layers IV and V, with a small number of cells detected in layers II/III and VIa^40,41^. Figure 2d shows the largest sagittal plane of a SOM-IRES-Cre:Ai3-EYFP mouse brain including almost all major brain regions. This result also demonstrated the data integrity of the whole-brain dataset and the variable density of SOM neuron distribution in different brain regions. Enlarged views of white square boxes in Fig. 2d illustrated that BPS enabled imaging of the entire brain in high enough detail to distinguish individual cell bodies in brain regions with variable cell densities (insets in Fig. 2d). These results demonstrate the ability of BPS to visualize single neurons for cell localization and counting in entire brains.

### Stereological cell counting

**Figure 3:**
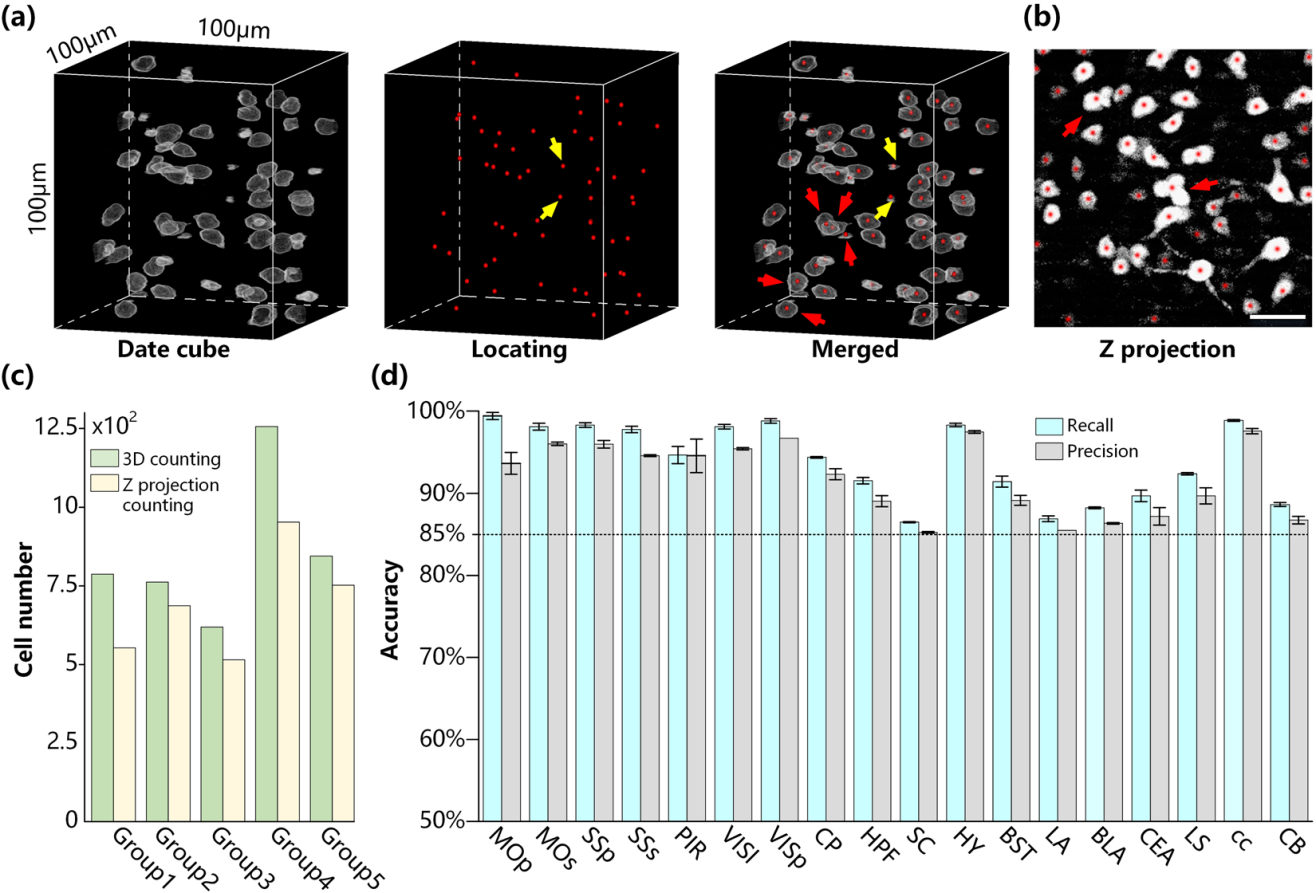
Stereological cell counting accuracy using the NeuroGPS algorithm. (**a**) Automatically locating the neurons in an SC data cube from a SOM-IRES-Cre:Ai3-EYFP mouse brain dataset using NeuroGPS. Grey represents EYFP-labelled somas in the raw data, and red dots indicate identified soma centres using the NeuroGPS algorithm. Overlapping grey somas and red dots indicate the correct identification of neurons. Yellow arrows represent erroneous commission. (**b**) The 100 μm-thick Z-projection image of the data cube in (**a**). Red arrows represent some disjunctive cells in the z-direction in (**a**) that partially overlap in (**b**). (**c**) Comparison between stereological and planar cell counting in 3D data cubes and their own z-projection images, respectively. (**d**) Accuracy of automated stereological cell counting in the regions of SOM-IRES-Cre:Ai3-EYFP mice brains (n = 3). Abbreviations: MOs, Secondary motor area; LS, Lateral septal nucleus; BST, Bed nuclei of the stria terminalis; cc, Corpus callosum; PIR, Piriform area; SSp, Primary somatosensory area; SSs, Supplemental somatosensory area; BLA, Basolateral amygdala nucleus; LA, Lateral amygdala nucleus; VISp, Primary visual area; VISl, Lateral visual areas; HY, Hypothalamus; CEA, Central amygdala nucleus; SC, Superior colliculus; CB, Cerebellum.

Accurately and automatically segmenting, locating and counting cell bodies is a prerequisite for quantifying the whole-brain cell distribution. To validate the accuracy of stereological cell counting, we compared NeuroGPS cell detection with manual identification (Fig. 3). Figure 3a depicts cell localization in a 100 × 100 × 100 μm data cube in the superior colliculus (SC) from the SOM-IRES-Cre:Ai3-EYFP mouse brain dataset. A total of 59 soma centres were automatically identified (indicated by red dots) using NeuroGPS, whereas 57 neurons were manually distinguished. Two background signals were mistakenly identified as neurons (indicated by yellow arrows in Fig. 3a). The recall and precision of automatic localization were calculated as 100% and 96.7%, respectively, for the overlap of automatically identified and manually detected cell bodies. We also generated a maximum intensity projection of this data cube to simulate traditional cell quantification in histological tissue sections (Fig. 3b). Due to no axial resolving power, some disjunctive but aligned cells in the axial direction of the data cube (indicated by red arrows in Fig. 3a) were partially overlapping in the projection image (indicated by red arrows in Fig. 3b), resulting in segmentation difficulties and potential counting errors. We compared the accuracy of stereological counting in the full volume and planar cell counting in the z-projection image of five randomly selected data cubes (Fig. 3c). The results showed that the cell numbers obtained by counting in 3D full volumes were higher than those obtained by counting z-projection images. We performed a Wilcoxon signed-rank test and acquired a two-sided P value was 0.043, demonstrating a significant difference between 3D counting and Z-projection counting. This result indicates potential erroneous omission resulting from the overlap of individual neurons along the z-axis, demonstrating that automated stereological cell counting is the only adequate method for unbiased cell quantification in a whole brain. Furthermore, we also estimated the accuracy of automated cell counting in 18 SOM-expressing brain regions from the three datasets (Fig. 3d). We randomly selected a representative volume of 512 × 512 × 512 μm in each region in each brain. The highest and lowest correct identification recall rates in these regions were 100% and 86.5%, respectively. The highest and lowest precision rates for correct identification in these regions were 100% and 85.2%, respectively. All three datasets exhibited identification rates higher than the standard of 85.0%. Average whole-brain recall and precision rates were 94.0 ± 0.4% and 91.8 ± 0.5% (n = 3), respectively. Furthermore, we randomly selected 20 neurons in each data cube and calculated their cell brightness. Pairwise comparisons between different datasets shows that there was no significant difference in the averaged cell brightness (p > 0.5 for all comparisons). It demonstrates slight brightness changes due to individual difference have no effect on cell counting. These results illustrated that stereological cell counting in whole-brain datasets using the NeuroGPS algorithm can accurately detect and segment soma as a potential method for quantifying brain-wide cell distribution.

### Brain-wide cell distribution of SOM neurons

**Figure 4:**
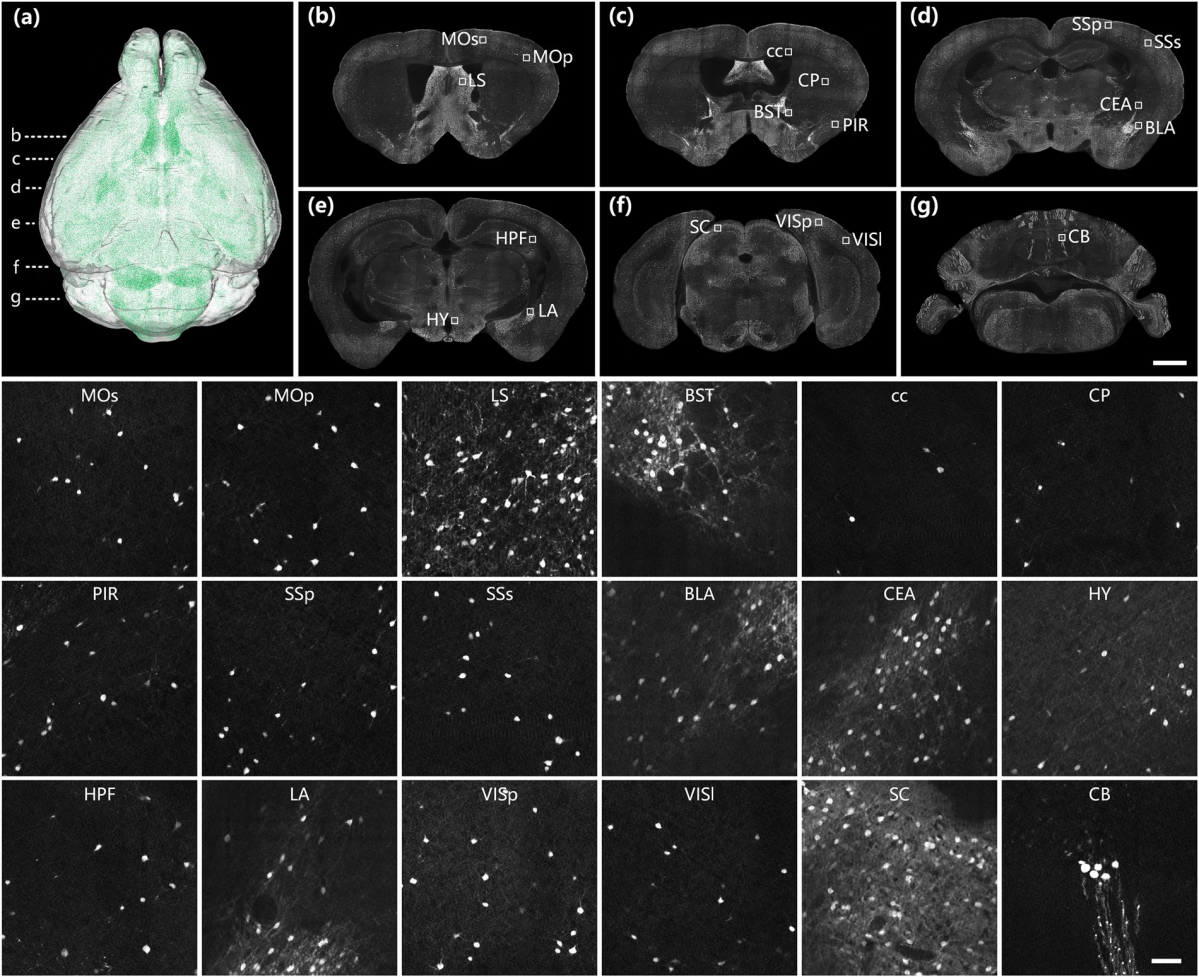
Brain-wide distribution of SOM-expressing neurons. (**a**) 3D volume rendering of the whole-brain dataset of a SOM-IRES-Cre:Ai3-EYFP mouse. Green represents the brain-wide expression of SOM. Dashed lines indicate the locations of images shown in (**b**–**g**). (**b**–**g**) A series of representative coronal images showing the distribution of SOM neurons in selected brain regions at a resampling resolution of 1 × 1 × 2 μm. The insets show corresponding partial enlarged images in (**b**–**g**) at the original resolution of 0.32 × 0.32 × 2 μm. Scale bar: (**b**–**g**) 1 mm and (insets) 50 μm.

Figure 4 shows the typical distribution of SOM-expressing neurons in a mouse brain. SOM was ubiquitously expressed in the brain, as shown in the volume rendering in Fig. 4a, in brain regions consistent with previous studies^32,35^. Figure 4b–g show a series of fluorescently labelled representative coronal plane images from the same dataset, including main SOM-expressing brain regions. The insets of the enlarged views of these regions demonstrated that different brain regions exhibit different densities of SOM-expressing neurons. SOM expression was densest in SC in the brain stem^32^. SOM was also relatively highly expressed in cerebral nuclei in the LS, CEA and BST regions, whereas SOM neurons were widely distributed with relatively low densities in the MOp, MOs, SSp, SSs, PIR, VISp, VISl and HPF regions of the cerebral cortex, the BLA and LA in the cortex subplate and the HY in the brain stem. There were relatively few SOM neurons in the CP in the brain stem and cc, although some densely arranged SOM neurons were observed in the CB. Some of this distribution variability within the SOM population in different regions might be functionally significant. These results provide a distribution reference for the functional study of SOM-expressing neurons.

### Morphological reconstruction of SOM neurons

**Figure 5:**
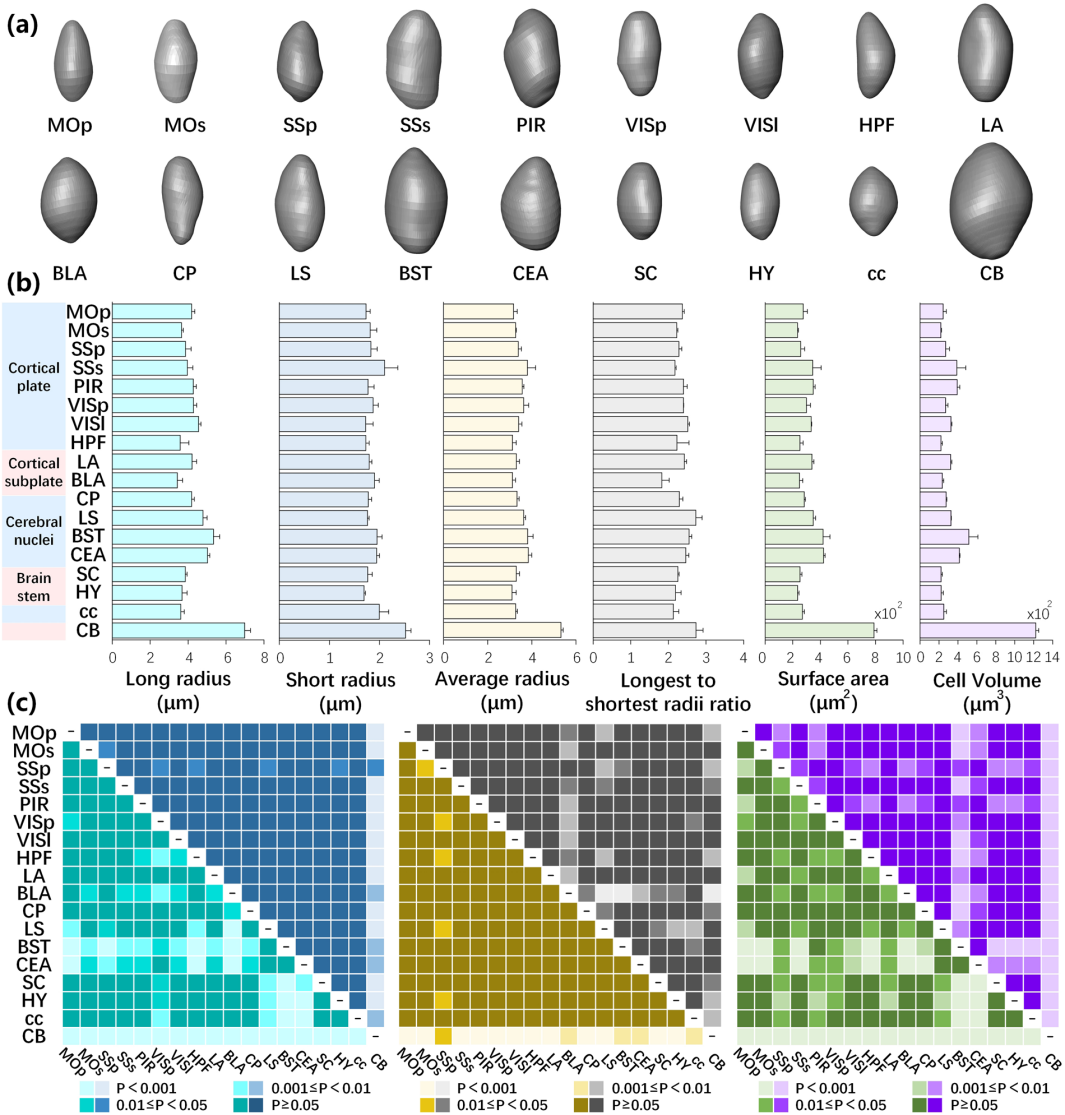
Morphological features of SOM neuronal somas. (**a**) Morphological reconstructions of typical SOM neuronal somas in specific brain regions. (**b**) Morphological features of SOM neuronal somas in spe cific brain regions in a data cube of 100 × 100 × 100 μm (n = 3). Error bars represent SEM. (**c**) Statistical significance of morphological features of SOM neuronal somas between different regions. Corresponding to (**b**), cyan, blue, yellow, grey, green and purple represent the long, short and average radii, longest to shortest radii ratio, surface area and cell volume, respectively.

Soma segmentation not only provides the central location of the cell body but also morphological characteristics of neuronal somas. We observed that SOM-expressing neurons in the same nuclei had similar morphologies, which might be used to classify subtypes of SOM-expressing neurons. We selected and reconstructed a typical 3D morphology of SOM-expressing neurons at the original resolution in 18 brain regions (Fig. 5a). Most cell bodies showed an ovoid or spindle profile with various cell volumes. SOM neuronal cell bodies in the CB were obviously bigger than all of the other somas. Furthermore, we randomly selected a data cube of 100 × 100 × 100 μm in each brain region, measured the longest and shortest radii, surface area and cell volume of all fluorescently labelled somas in the data cube, and calculated the corresponding average radius and longest to shortest radii ratio (Fig. 5b). The results demonstrated that both the longest and shortest radii and the surface area and cell volume of SOM-expressing neurons in the CB were higher than in other regions. However, the longest to shortest radii ratios for neurons in the CB were similar to those in other regions. This result indicated that SOM-expressing neurons had similar soma profiles in different regions, consistent with the reconstructions shown in Fig. 5a. Except for the CB, SOM-expressing neurons in other regions exhibited similar ranges of shortest radii and various longest radii, demonstrating similar characteristics for average radii and the longest to shortest radii ratio. The longest and shortest radii in BST and CEA were overall longer, thus the corresponding surface area and cell volume were bigger. The morphological features of SOM-expressing neurons in the neocortex were relatively consistent. We also performed a statistical analysis on the morphological traits of SOM neuronal somas (Fig. 5c). There was no significant difference in the soma morphological features for the pairwise comparisons between most regions. Some regions presented differently compared with other regions. For example, CB showed a significant difference in most morphological parameters with other regions, except the longest to shortest radii ratio. It indicates that combing physiological experiment and gene expressing measurement in the future might distinguish if SOM neurons in the CB belong to specific subtype of SOM neurons. These results illustrated that the platform used in this study enabled the accurate reconstruction and extraction of morphological features for cell type studies.

### Quantifying the whole-brain distribution of SOM neurons

**Figure 6:**
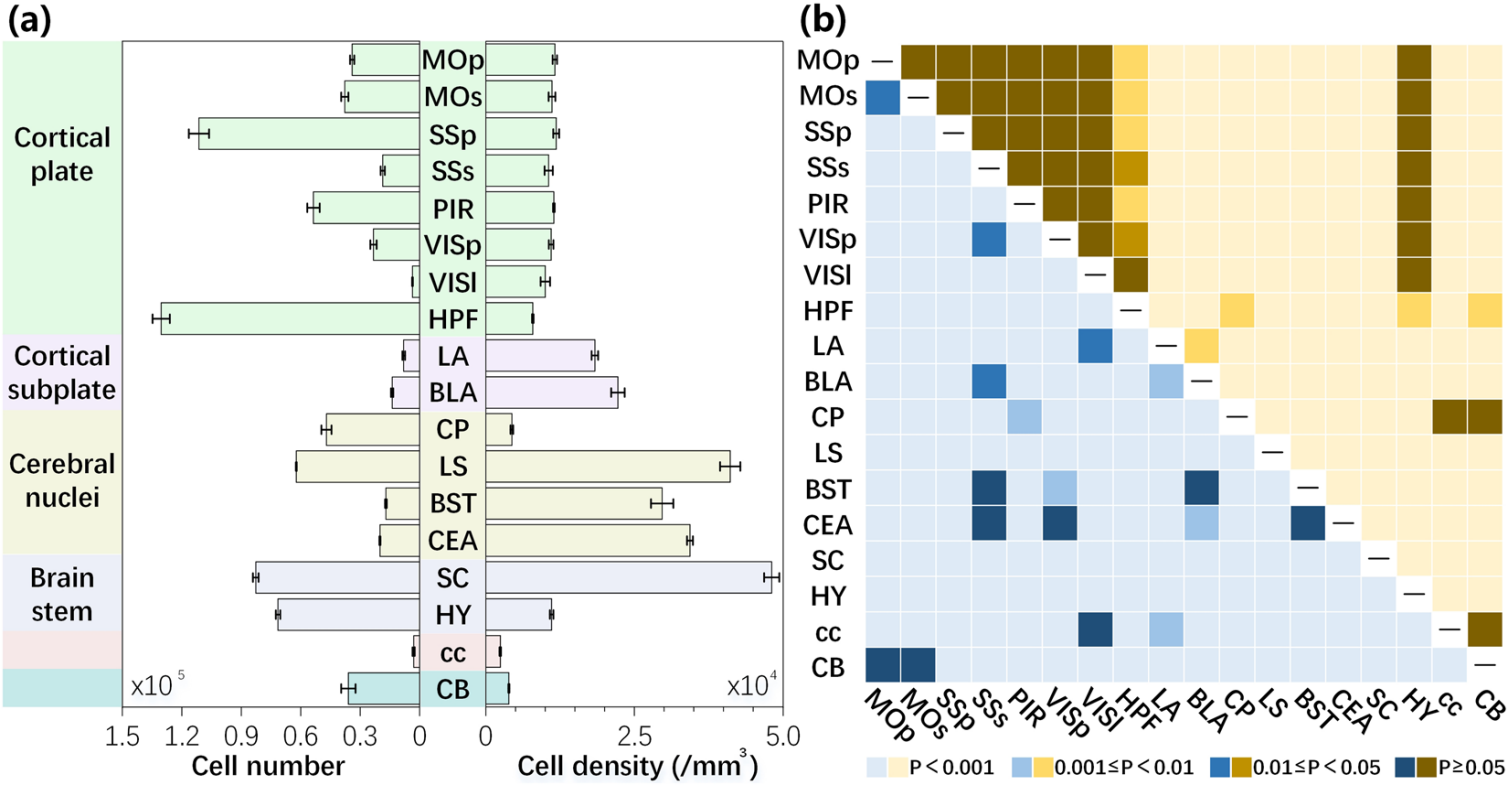
Quantitative analysis of the whole-brain distribution of SOM-expressing neurons (n = 3). (**a**) Cell numbers and densities in main brain regions. Error bars represent SEM. (**b**) Statistical significance between different regions. Blue and yellow represent cell number and density, respectively.

Quantitative mapping of a given type of neuron in the entire brain can improve our understanding of this neuron type in specific brain functions. To explore this diversity, we comprehensively quantified the distributions of SOM-expressing neurons in each region of three brains (Fig. 6). The total number of SOM-expressing neurons in a SOM-IRES-Cre:Ai3-EYFP mouse brain was 1,901,273 ± 67,096 (Fig. 6a). In terms of numbers, the most prominent labelling was observed in the HPF (130,431 ± 2,531 cells) due to its large volume, with denser labelling than reported in a previous study^34^. The numbers in the SSp were also higher than in other regions (111,365 ± 2,965 cells). SC, HY, LS and PIR showed 5~9 × 10^4^ SOM-expressing neurons, whereas the CP, MOs, CB, MOp, VISp, CEA, SSs, BST and BLA have 1~5 × 10^4^ SOM-expressing neurons. LA, VISl and cc exhibit less than 1 × 10^4^ SOM-expressing neurons. There were only 2,996 ± 29 SOM-expressing neurons in the cc. Cell density showed distinct diversity in terms of cell number, reflecting a volumetric difference in various brain regions. The qualitative impression of the brain-wide distribution patterns was supported after calculating the cell density. Across the whole brain, the most abundant labelling was found in the SC, LS and CEA, consistent with Fig. 4. The cell densities in these regions were 48,130 ± 1,278, 41,140 ± 1,708 and 34,370 ± 498 cells/mm^3^, respectively. The regions in the neocortex had similar cell densities, consistent with previous studies^32^, and the cells were much denser than the 2D density reported in a previous study^39^. The average cell density in the neocortex was 10,735 ± 422 cells/mm^3^. The cell density of HY in the brain stem was similar to the neocortex. CP and CB showed low SOM expression densities of 4385 ± 233 and 3882 ± 91 cells/mm^3^, respectively, reflecting their large volumes. SOM was sparsely expressed in the cc (2431 ± 116 cells/mm^3^). We also compared the cell distributions between different regions (Fig. 6b). Different from the statistical results of the soma morphology (Fig. 5c), both cell number and density presented a significant diversity among different regions. There was a significant difference in the cell number for the pairwise comparisons of most regions. For cell density, there was no significant difference for the pairwise comparisons of sub-regions of cortex, cortex and HY, CP, cc and CB. The other pairs presented differently. This distribution information could potentially be used as a reference for studying SOM neurons, and these results demonstrated that this platform could be used for the quantitative analysis of cell distribution in the whole brain.

## Discussion

Understanding the principles of brain functions requires the systematic characterization of participating cell types. Establishing methods to efficiently identify and quantify specific neurons will facilitate our understanding of how neurons are involved in specific functions. Advances in whole-brain optical imaging and stereological cell localization provide a method to answer fundamental questions concerning cell distribution and cell number. BPS enabled the acquisition of fine structural information with the co-located anatomical reference of labelled neurons in the whole brain at subcellular resolution. NeuroGPS is capable of automatically detecting, locating and counting cell bodies in 3D. In this study, we combined these two technologies to constitute a novel platform to quantify a comprehensive distribution of type-specific neurons. To demonstrate the utility of this platform, we obtained high-resolution brain-wide datasets of SOM-IRES-Cre:Ai3-EYFP mouse brains with co-located cytoarchitecture and quantitatively analysed the distribution features of SOM expression and the morphological features of SOM neural somas. The results provide a platform with the capability of precise orientation, fine soma morphological reconstruction and accurate cell counting of a given type of neuron in the whole brain.

The automatic whole-brain optical imaging and stereoscopic cell counting and reconstruction demonstrated an unprecedented ability to accurately and quantitatively detect and analyse the distributional features of specific neurons in the whole brain. Acquiring a 3D brain-wide structural dataset is a prerequisite for accurately locating and counting the cells throughout the whole brain in the stereological approach. In conventional 2D approaches, information overlap between axial projections easily leads to cell omission and increases the difficulty of segmenting and counting. Interval sampling decreases the workload of manual data acquisition but fails to depict the real topological organization of the neurons. In contrast, visualizing neurons with full-volumetric whole-brain imaging overcomes these limitations and records realistic brain-wide cell organization in a reliable and efficient manner. Whether or not to precisely segment axial-aligned or adjacent somas in 3D is the key factor influencing automatic locating and counting accuracy, and this step requires micron- or even submicron-scale imaging resolution in the whole brain, although the sizes of neuronal somas are at the scale of tens of microns. The strategies of combing optical imaging and mechanical sectioning for whole-brain imaging^25–27,42^ have the potential to meet this requirement. The results of this study demonstrated this technical advantage over conventional 2D methods. Based on the high-resolution whole-brain 3D dataset, stereoscopic cell counting by NeuroGPS avoided the missed counting of axially overlapped cells and possible repeat counting of cells in adjacent slices.

Furthermore, this platform takes advantage of accurate neural morphological reconstruction with high resolution. Characterizing a cell type requires cross-validating the molecular, anatomical and physiological proerties^43^. Previous studies demonstrated the predominant cell types of SOM neurons were Martinotti cells (MCs) in the neocortex and oriens-lacunosum moleculare (O-LM) neurons in the hippocampus^34,35,40,44^. Most morphological works focused on soma location, dendritic morphology, axonal tree pattern and neuronal connectivity. Wang, Y. *et al*.^40^ performed a systematic multidimensional study of the detailed anatomical, physiological and molecular properties of MCs in the cortex. They found most MC somas in the cortex were ovoid or spindle shaped, consistent with our results. Figure 5 shows SOM neurons in most regions except CB had a similarity of the soma morphology. The significant difference of SOM neuronal somas between CB and other regions indicates that further molecular and physiological study might be considered to distinguish potential different subtypes. In addition, the digital reconstruction of individual soma is suitable for the quantitative analysis and computational modelling of neuronal cell body shape changes during developmental stages and pathological conditions.

Generating a comprehensive database of the distribution and morphology of type-specific neurons elucidates the role of neurons in neural circuits and informs the design of experiments to study their function. The regional diversity of SOM expression in this study provides an important anatomical reference for SOM-related specific functions. This platform enabled the precise regional analysis of a given type of neuron, reflecting the advantage of simultaneously acquired cytoarchitecture in the same brain with BPS. Regional distribution of soma locations indicates further detailed morphological analysis of dendritic and axonal arbours for future cell type study^40^. Using this platform, researchers can study morphological and distributional alterations of neuronal somas during developmental and evolutionary processes. This platform also facilitates the investigation of the neuronal loss and death in neurodegenerative and psychiatric disease progression^9^. We propose that this platform could potentially become a routine tool in neuroscience research.

## Methods

The pipeline for quantifying the cell distribution of type-specific neurons in the whole mouse brain consisted of five steps. (1) Labelling the type-specific neurons in the whole brain using genetic or viral approaches. (2) Imaging the whole brain at subcellular resolution by BPS. (3) Registering with the reference atlas and segmenting brain regions. (4) Segmenting, localizing and counting cell bodies in 3D by NeuroGPS. (5) Statistically analysing the cell distribution and morphological features of specific regions.

### Animals

The SOM-IRES-Cre transgenic mouse line^32^ was obtained from Josh Huang’s lab (Cold Spring Harbor Laboratory, Cold Spring Harbor, NY, USA) to genetically target SOM neurons. The Cre-reporter expressing EYFP Ai3 mice (JAX no. 007903) were purchased from Jackson Laboratories. To fluorescently label SOM-INs, heterozygous SOM-IRES-Cre:Ai3-EYFP mice were obtained by crossing SOM-IRES-Cre and Ai3-EYFP mice. Three 12-week-old adult SOM-IRES-Cre:Ai3-EYFP mice were used. The experiments were performed in mice of either sex. Three 16-week-old SOM-IRES-Cre:Ai3-EYFP male mice was used for immunostaining. The mice were housed on a 12-h light/dark cycle with food and water ad libitum. All of the animal experiments were approved by the Institutional Animal Ethics Committee of Huazhong University of Science and Technology and all experiments were performed in accordance with relevant guidelines and regulations.

### Resin embedding

All of the histological procedures have been previously described^45^. Briefly, SOM-IRES-Cre:Ai3-EYFP mouse were anaesthetized with a 1% solution of sodium pentobarbital and subsequently intracardially perfused with 0.01 M PBS (Sigma-Aldrich Inc., St. Louis, MO, USA), followed by 4% paraformaldehyde (Sigma-Aldrich Inc., St. Louis, MO, USA) and 2.5% sucrose in 0.01 M PBS. The brains were excised and post-fixed in 4% paraformaldehyde at 4 °C for 24 h. After fixation, each intact brain was rinsed overnight at 4 °C in a 0.01 M PBS solution and was subsequently dehydrated in a graded ethanol series (50%, 70%, and 95% ethanol at 4 °C for 1 h each). We modified the previous resin-embedding approach to reduce background fluorescence. Briefly, after dehydration, the brains were immersed in a graded series of GMA (Ted Pella Inc., Redding, CA, USA) including 0.2% Sudan Black B (SBB): 70%, 85%, and 100% GMA for 2 h each and 100% GMA overnight at 4 °C. Subsequently, the samples were impregnated in a prepolymerized solution of GMA for 3 days at 4 °C and finally embedded in a vacuum oven at 48 °C for 24 h. The 100% GMA solution contains 67 g A solution, 2.8 g deionized water, 29.4 g B solution, 0.2 g SBB, and 0.6 g AIBN as initiator. The 70% and 85% GMA (wt/wt) were prepared from 95% ethanol and 100% GMA.

### Immunostaining

Post-fixed brains were sectioned at 50 µm thickness using a Leica vibratome (Leica VT1200S, Germany). Sections were permeabilized using 0. 3% Triton-X 100 in PBS and blocked with 5% bovine serum albumin (BSA, Sigma) and 0.3% Triton in PBS and subsequently incubated with anti-SOM (goat polyclonal; 1:300; Millipore) at 4 °C overnight. The sections were washed three times for 10 min in 0.1 M PBS at room temperature and incubated with secondary antibodies (Alexa Flour 594, Rb-Anti-Gt-g(ab’)2,H, Invitrogen) for 2 h at 37 °C. The sections were washed three times for 10 min in 0.1 M PBS at room temperature. The sections were mounted and coverslipped with Fluorogel. The representative images were acquired with a Zeiss 710 LSM confocal microscope (Zeiss, Jena, Germany).

### Whole-brain imaging

We performed the data acquisition with BPS^27^. Briefly, we fixed the sample on a high-precision 3D translation stage. During data acquisition, the sample was immersed in a 2 μg/ml propidium iodide (PI) solution to counterstain the cytoarchitecture of the sample in real time during data acquisition. We used two-channel structured illumination microscopy (SIM)^46^ to simultaneously image EYFP-labelled SOM neurons and PI-counterstained cytoarchitecture at a voxel resolution of 0.32 × 0.32 × 2 μm. Optical sectioning of SIM enabled the acquisition of the image slightly below the top surface to avoid the effect of sectioning marks. Mosaic scanning was performed to acquire the entire coronal image of the sample surface. There was an overlap of 12 pixels between adjacent mosaics for lateral registration. Subsequently, the sample on the stage was moved to a diamond knife to remove imaged tissue. This imaging-sectioning-staining cycle was repeated until the entire sample was imaged. The total data acquisition time for each brain was approximately 130 h. The whole-brain datasets had a size of approximately 4 terabytes, including approximately 5400 coronal images, for each channel.

### Segmenting brain region

We identified anatomical brain regions based on the Allen mouse Reference Atlas^47^ using PI-counterstained signals. We downsampled the original data set at the voxel resolution of 3.2 × 3.2 × 3.2 μm and rotated and translated the data for 3D spatial correct to easily figure out the corresponding coronal planes in the reference atlas. Subsequently, we resampled the transformed data of PI-stained images into 3.2 × 3.2 × 20 μm and depicted the contours of each region using Amira software (v 5.2.2, FEI, Merignac Cedex, France). The segmentation editor module of Amira was utilized for the manual outline segmentation of the whole mouse brain. Referring to the cytoarchitecture of each region in the Allen mouse Reference Atlas^47^, we manually identified the contour of each region in the PI-stained cytoarchitecture. The outlined contour was saved as a TIFF file.

### Localization and counting

We used the NeuroGPS algorithm^28^ to automatically segment, locate and count somas in 3D space. With the NeuroGPS algorithm, we employed binarization and erosion operations to extract the foreground and subsequently used an L1 minimization model^48^ to locate the somas in the extracted regions. Limited by the memory capability of the graphic workstation (T7600 with two Intel E5–2687w CPUs, 256 GB memory and an Nvidia K6000 graphics card, Dell Inc., Round Rock, Texas, USA), each dataset of EYFP-labelled SOM neurons was divided into 50,000 blocks of 512 × 512 × 512 μm with a resolution of 1 × 1 × 2 μm. The NeuroGPS analysis was performed in the graphic workstation cube by cube to identify all of the EYFP-labelled neurons. The cell locations and numbers of SOM neurons in the whole brain were collected and saved as an SWC file for each brain.

To evaluate the counting omission and commission errors made by NeuroGPS, we compared the results of automatic and manual recognition. Ground truth was back-to-back validated by three skilled researchers. B1 and B2 indicate the total neuronal numbers by manual and automatic recognition, respectively, whereas B indicates the cell number using both approaches. The recall R is defined as R = B/B1, whereas the precision P is defined as P = B/B2. We randomly selected 5 data cubes of 512 × 512 × 512 μm from a whole-brain dataset and generated 10 images of 50 μm-thick z-projection for each volume. Subsequently, we performed stereological and planar cell counting in full volumes and z-projection images, respectively, and calculated the R and P for each data cube.

### Reconstruction and visualization

We reconstructed and visualized the cell body morphology of SOM neurons in the midsagittal plane of the brain using Amira software. The process included extracting the data in a range of interest, sampling or interpolation, reslicing the images, identifying the maximum intensity projection, and volume and surface rendering using the main Amira modules.

### Statistical analysis

We compared the morphological features of SOM neuron somas. We measured the longest and shortest radii, surface area and cell volume of the SOM neuron somas with a resolution of 0.32 × 0.32 × 2 μm by NeuroGPS. We also analysed the SOM distribution of each region by comparing the cell number and density of each region. The corresponding SWC files were recalled according to the 3D contour of each region in the PI-stained signal and merged together as the counting result of each region. The volume for each region was measured according to the 3D contour of each region for the PI-stained signal in the Amira software program. Subsequently, we divided the cell number of the brain region by the corresponding volume to get the cell distribution density for the region. This process was performed in all three whole-brain datasets. All of the results are presented as the MEANs ± SEM. Moreover, we assessed the statistical significances of soma morphology and cell distribution between different regions using analysis of variance (ANOVA). We also statistically compared stereological and planar cell counting using Wilcoxon signed-rank test. We performed these statistical calculations using SPSS Statistics (v 22, IBM, New York, USA). P values <0.05 were considered significant.
